# Occlusive and Non-Occlusive Application of Microemulsion for Transdermal Delivery of Progesterone: Mechanistic Studies

**DOI:** 10.3797/scipharm.1201-01

**Published:** 2012-06-18

**Authors:** Gamal M. El Maghraby

**Affiliations:** Department of Pharmaceutical Technology, College of Pharmacy, University of Tanta, Tanta, Egypt.

**Keywords:** Nanoemulsion transdermal, Supersaturation, Ethanolic microemulsion, Open application, Phase behaviour

## Abstract

This work evaluated the occlusive versus non-occlusive application of microemulsion (ME) for the transdermal delivery of progesterone. The mechanisms of enhanced skin penetration were investigated. ME comprised of oleic acid, Tween 80, propylene glycol, and water, was used neat or with ethanol as a volatile cosurfactant. The ME formulations enhanced progesterone transdermal flux compared to the saturated drug solution in 14% aqueous propylene glycol (control). Ethanol-containing ME (EME) was better than the ethanol-free system (EFME). Open application of EFME produced a marginal reduction in flux compared to occlusive application. For EME, open application reduced the flux by 26–28% with the flux remaining significantly higher than that obtained with EFME. The mechanistic studies revealed synergism between ethanol and EFME with EME, producing greater flux than the sum of fluxes obtained from 40% ethanol in water and EFME. Penetration enhancement and supersaturation played a role in enhanced transdermal delivery, but other mechanisms were also possible. This study thus introduced EME as a transdermal delivery system for progesterone with good potential for open application as a spray.

## Introduction

The use of microemulsions as skin drug delivery systems has recently gained interest. Microemulsions are thermodynamically stable, transparent, single-phase, optically isotropic liquid systems of water, oil, and surfactants [1]. They can be considered as ideal liquid vehicles for drug delivery as they have most of the requirements for this including the thermodynamic stability, ease of formulation, low viscosity, high drug-loading capacity, and small droplet size. The latter characteristic provides better chance for contact and adherence to skin, thereby transporting drugs in a controlled manner [2, 3]. In addition, the components of microemulsions can be selected to produce a penetration-enhancing effect, providing additional advantages for their application in transdermal drug delivery. Numerous reports have been published indicating the potential of microemulsions for the enhanced drug delivery of different drugs [4–6]. The efficacy of microemulsion depends on the components which comprise the oil, the surfactant, and the cosurfactant. Oils with a stronger penetration-enhancing effect were superior [7]. Cosurfactants were shown to have a significant effect with ethanol, being superior to other volatile or non-volatile cosurfactants [6, 8]. The mechanism of enhanced transdermal drug delivery from microemulsions is controversial, with alternative strategies being hypothesized. The suggested mechanisms include the high-solubilizing power of microemulsion and the absorption-enhancing effect of the microemulsion components [2, 9]. The ability of microemulsion ingredients to enter the skin with the result of increased drug partitioning into the skin, was reported as another possible strategy [2]. The possibility of direct drug transfer from the microemulsion droplet to the stratum corneum was also suggested [8]. The microstructure of the microemulsion system can create a very large surface area for drug transfer to skin. The very small droplet size and very low interfacial tension of microemulsion can provide excellent surface contact with the skin surface. This will allow the vehicle to fill even the microscopic gaps on the skin’s surface. This was considered to be an explanation for enhanced skin-drug transfer [6]. The last possible mechanism depends on the supersaturation process, which increases the thermodynamic activity and the driving force for transdermal drug transfer [10, 11]. It should be noted that the proposed mechanisms depend on the fluidity of the microemulsion system and may be influenced by the method of application.

Despite encouraging reports on the use of microemulsions for enhanced transdermal drug delivery, none of the studies investigated the effect of the application method of micro-emulsions on their efficacy. The investigators employed mainly occlusive application of the fluid system. This application protocol may not be easy for fluid systems in clinical practice. Alternatively, fluid systems are better employed by an open application protocol through the formulation of spray [12]. The open application is always associated with evaporation of the volatile components of the microemulsion, resulting in phase transition, which may create systems with varying thermodynamic activity and may lead to supersaturation, an effect which requires investigation. In addition, the volatile components may have penetration-enhancing effects which can be diminished upon evaporation. Accordingly, the objective of this work was to investigate the effect of the method of application on the efficacy of microemulsion as a skin drug delivery system. The study was extended to mechanistic investigations. The mechanistic studies probed the influence of supersaturation and the penetration-enhancing effect of the volatile components. The synergistic effect of the formulation components was also monitored. To achieve these goals, progesterone was selected as the model drug. A formulation containing oleic acid with Tween 80/propylene glycol and water was used as the basic ethanol-free microemulsion. This system was mixed with increasing concentrations of ethanol to produce ethanol-containing microemulsions. Propylene glycol was included in the basic microemulsion to provide an antinucleant effect after evaporation of ethanol as a result of open application.

## Results and Discussion

### Pseudo-ternary phase diagrams

**Fig. 1:**
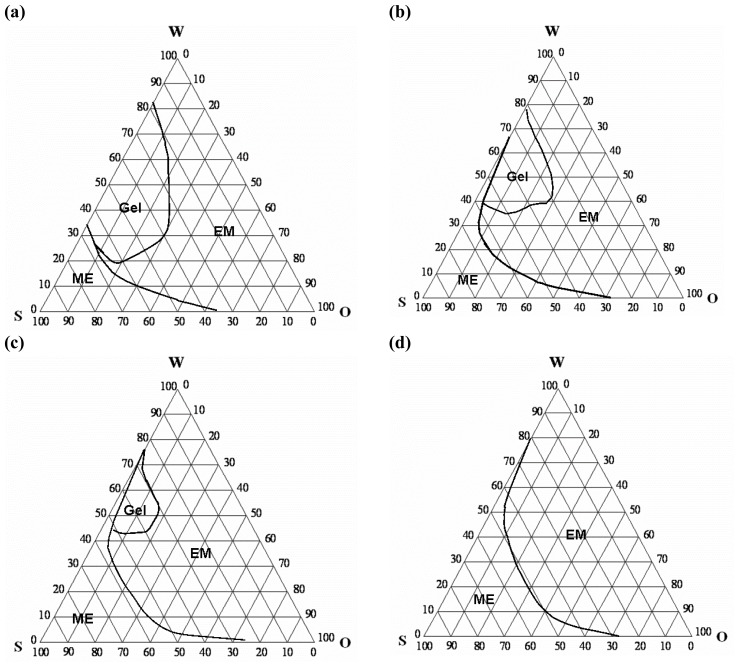
Pseudo-ternary phase diagrams of oleic acid in the presence of different surfactant/cosurfactant systems. (a) is a Tween 80-based system, (b) is Tween 80 – propylene glycol (4:1), (c) is Tween 80 – propylene glycol – ethanol (4:1:1.8) and (d) is Tween 80 – propylene glycol – ethanol (4:1:5). ME = microemulsion and EM = coarse emulsion.

The current study investigated the transdermal delivery of progesterone from oleic acid-based microemulsions (ME). Tween 80 was used as the surfactant and propylene glycol (PG) was the cosurfactant. To investigate the influence of volatile components on transdermal delivery from ME after occlusive and open application, ethanol-containing ME was employed. The presence of ethanol and/or PG can affect the phase behaviour of the system [6]. Accordingly, pseudo-ternary phase diagrams were constructed in the absence and presence of PG with and without increasing concentrations of ethanol. These phase diagrams are shown in Fig. 1. The simplest phase diagram (Fig. 1a) was constructed using oleic acid as the oil, Tween 80 as surfactant, and water. This system produced a phase diagram in which the ME zone occupied approximately 15% of the total area of the phase diagram. Moving towards the border between the ME and other phases, the system tends to be more viscous and transforms to a gel or coarse emulsion. The gel phase occupied approximately 20% of the total area of the phase diagram (Fig. 1a).

The incorporation of cosurfactants increased the area occupied by the ME zone. Incorporation of PG as a cosurfactant (4:1; Tween/PG) increased the area occupied by the ME zone to 17%, and reduced the gel phase to approximately 12% (Fig. 1b). Addition of ethanol to this system to provide a surfactant-cosurfactant mixture of Tween 80, PG, and ethanol (4:1:1.8) resulted in a further increase in the area occupied by the ME zone to reach 21%. This effect was accompanied with a significant reduction in the gel phase to occupy only 5% of the total area of the phase diagram. Increasing ethanol concentration in the surfactant-cosurfactant to produce a system of Tween 80, PG, and ethanol (4:1:5) increased the area occupied by the ME zone to reach approximately 27%. This effect was associated with abolishing the gel phase.

The presence of PG or ethanol in the ternary systems was previously shown to increase the existence of ME and break the gel and liquid crystalline structures [6, 13]. The presence of PG and/or low concentration of ethanol in the current study increased the ME zone, but failed to completely break the gel structure. The latter can be explained on the basis of the low concentration of PG, and that the ethanol was not sufficient to destroy the gel structure. A higher concentration of ethanol abolished the gel structure. These results indicate that the concentration of cosurfactant is a determining factor in the phase behaviour of the ternary system.

The formation and range of the microemulsion zone depend on the physicochemical properties of the oil phase, aqueous phase, and surfactant. The very low interfacial tension, the presence of the highly fluid interfacial film of surfactant, and the association of oil molecules with the interfacial surfactant film are other essential parameters affecting the formation of ME [14]. The mechanism of action of cosurfactants can be attributed to the reduction of the surface tension and fluidization of the interfacial surfactant film [15, 16].

### Characterization of the tested formulations

Table 1 presents the characterization of the tested formulations. The saturation solubility of progesterone in ME formulations revealed high-solubilizing power of the ME systems, compared to that of the simple hydroalcoholic or aqueous propylene glycol systems. The solubilizing power of ME increased significantly after incorporation of ethanol (Table 1). The high-solubilizing power can be due to the cosolvency of the ME components with a significant contribution to the small droplet size, which provides a massive surface area for drug incorporation. This explains the increased solubilizing power of ME in the presence of ethanol, which is believed to reduce further surface tension and decrease the droplet size. The recorded solubility of the drug in 14% PG in water (PGW) can be correlated to the previously recorded data [17].

### Ethanol evaporation

**Fig. 2:**
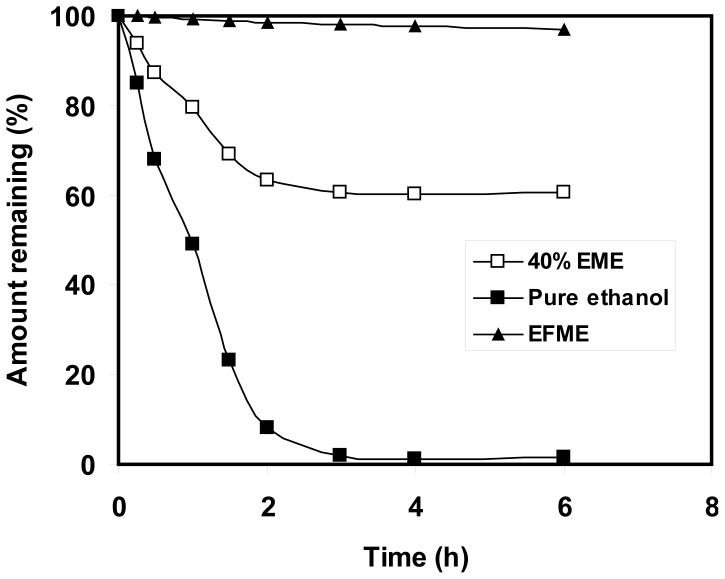
Evaporation profiles of ethanol-free (EFME) and ethanol-containing 40% EME) formulations. The evaporation profile of pure ethanol was calculated from the weight loss of 40% EME considering that the weight loss is due to evaporation of ethanol.

The ethanol evaporation profile from the 40% EME was monitored after incubation in a Petri-dish at 32 °C. This can approximately predict the evaporation profile of ethanol from the EME after open application to skin. The weight loss from the EFME was monitored and was used as the control. These profiles are shown in Fig. 2.

The ethanol-free formulation showed marginal loss after incubation at 32 °C, with the formulation losing about 2% in the first 3 hours (Fig. 2). The EME formulation lost about 37% in the first 2 hours, with the loss approaching 40% after 3 hours. Considering this with the marginal loss from EFME, the loss from 40% EME can be considered mainly due to ethanol evaporation. This indicates that most ethanol was evaporated from the formulation in the first 2-3 hours. Complete evaporation of ethanol was previously recorded after 2 hours [18]. This study indicated that open application of EME will lead to a loss of the ethanol content in the first 2–3 hours. However, it should be emphasized that this study is just an approximation of the skin permeation study, as in the real skin application part of ethanol will diffuse into and through the skin within the same period. This means that part of the ethanol can affect the natural barrier that the skin provides and influence the permeation process with the other part being evaporated after open application.

### Transdermal delivery of progesterone from ME: Occlusive versus open application

The tested ME formulations were comprised of oleic acid as the oil, Tween 80 as the main surfactant, with PG as the cosurfactant alone or with ethanol, as the volatile component. The concentration of PG in the ethanol-free ME (EFME) was 14% w/w. Accordingly, it was decided to employ a saturated drug solution in 14% PG in water as the control. Excess drug was included in this solution to maintain saturation throughout the permeation study. The occlusive application of this control provided the transdermal flux value of 1.09 μg cm^−2^ h^−1^ (Table 2). This flux is similar to that recorded from the saturated solution in 40% PG in water, but using porcine skin [17]. Open application of the control produced only a trend of increased transdermal progesterone flux (P > 0.05; Table 2). Occlusive application of EFME increased the transdermal progesterone flux by 2.5-fold, compared with the control. This increase is expected and complies with published findings [2, 6, 11]. Open application of EFME produced a marginal reduction in the transdermal drug flux (only 12% reduction).

Incorporation of ethanol in the ME formulations resulted in a significant increase in the transdermal progesterone flux, with the flux increasing upon increasing the ethanol concentration in the ME. Thus, occlusive application of 20% EME increased the transdermal flux by 5.7-fold compared to the control, and by 2.3-fold compared to the EFME (Table 2). Occlusive application of 40% EME further increased the transdermal progesterone flux, with the values being 9-fold compared to the control, and 3.7-fold compared to EFME (Table 2). The recorded increase in drug flux after application of ethanol-containing ME was accompanied by a reduction in the lag time compared to the EFME or control. This indicates an increased diffusivity of the drug after application of ethanol-containing ME.

The positive effect obtained after incorporation of ethanol in ME complies with the previously recorded findings [6]. Open application of ethanol-containing ME reduced the transdermal progesterone flux by 28 and 26% in cases of 20% EME and 40% EME, respectively. However, it should be emphasized that the recorded flux values remained higher than that obtained from EFME. Considering the ethanol evaporation profile after open application, which is approximately derived from Fig. 2, open application will lead to loss of the ethanol content within the first 2-3 hours after application. Nevertheless, the flux recorded after open application of ethanol-based ME was higher than that recorded after occlusive or open application of EFME. This may be explained on the basis of ethanol-induced penetration enhancement, which could have been performed before evaporation. Other explanations may include the effect of ethanol on the ME structure (resulting in reduced droplet size and lower surface tension) or the possible supersaturation, which can result from the evaporation of ethanol which increased the solubilizing power of ME. These explanations will be verified in the proceeding sections.

### Mechanisms of enhanced skin delivery of progesterone from ME after occlusive and open application

This section was conducted with the goal of explaining the findings recorded in the previous section. The main findings included the significant increase in the efficacy of ME after incorporation of ethanol, and the 26–28% reduction in transdermal flux obtained from ethanol-containing ME after open application compared to occlusive application of the corresponding formulation, with the flux obtained after open application remaining well higher than that obtained after application of the ethanol-free formulation.

To explain the first finding, the transdermal delivery of progesterone was studied from saturated aqueous solutions containing the ethanol (ETW) at the highest concentration included in ME (40%). Occlusive application of ETW produced a 2-fold increase in the transdermal flux of progesterone compared to the control (Table 2). This effect can be explained on the basis that ethanol can rapidly diffuse into and through the skin, perturbing its barrier nature and increasing both the partitioning and diffusion of the drug into and through the skin [19]. Open application of the same ethanolic solution (ETW) reduced the flux by 21% compared to occlusive application, but the flux remained higher than that obtained from the control (Table 2). This means that ethanol was able to produce some enhancing effect due to rapid diffusion before evaporation in the first 2–3 hours. Considering the flux obtained from 40% EME (9.83 μg cm^−2^ h^−1^), it was found to be higher than the sum of the flux values obtained from EFME (2.69 μg cm^−2^ h^−1^) and that obtained from 40% ethanol (2.22 μg cm^−2^ h^−1^). This means that the penetration-enhancing effect of ethanol is not the main factor responsible for the significant increase in the efficiency of ME after incorporation of ethanol. Thus, other factors must be considered in ethanol-enhanced efficacy of ME. These factors include the reduced droplet size, increased surface area, higher solubilizing power, reduced surface tension, and subsequently increasing the intimate contact between ME and skin. These factors can promote better drug permeation [2, 6].

To explain the data obtained after open application of ethanol-containing ME, after open application where the flux remained higher than that obtained after application of the ethanol-free formulation, two possibilities were investigated. The first is the penetration enhancement obtained from the rapid diffusion of ethanol, which can take place before complete evaporation. The second is the possibility of the in-situ supersaturation process, which can take place upon ethanol evaporation. The first possibility was tested by open skin pre-treatment with 40% ethanol before open application of EFME. The results are presented in Table 3. The data revealed a marginal increase in the transdermal progesterone flux after application of EFME to pre-treated skin, compared to that obtained after open application of the same formulation to non-treated skin. The flux was increased from 2.36 to 2.85 μg cm^−2^ h^−1^ after application to pre-treated skin. This was associated with a decrease in the lag time to reach 1.79 hours in the case of pre-treated skin compared to 3.41 in case of non-treated skin (Table 3). The flux recorded in the case of pre-treated skin was significantly lower than that obtained after application of ethanol-containing ME (40% EME, Table 2). This finding indicates that ethanol-induced penetration enhancement is not the main factor responsible for the superiority of open application of ethanolic ME over EFME.

To investigate the influence of supersaturation on the enhanced transdermal delivery of progesterone after open application of ethanol-containing ME, the transdermal delivery of the drug from ME containing the drug at 2 degrees of saturation (DS) was monitored. ME containing progesterone at 2 DS was prepared by formulating 40% EME containing 43.4 mg/ml of progesterone. This concentration is 60% of the saturation solubility of the progesterone in 40% EME, and double the saturation solubility of the drug in EFME. This formulation is assumed to supersaturate upon evaporation of ethanol after topical application (in-situ supersaturation). Based on the composition of this formulation, if supersaturation is not operating, we shall obtain a flux value equivalent to 60% of the flux recorded after open application of fully saturated 40% EME. A flux higher than expected can suggest the role of supersaturation. If supersaturation is the main mechanism, the recorded flux from ME containing 2 DS should be double that of the flux recorded after open application of EFME. The same principle was adopted previously for the enhanced transdermal delivery of lipophilic drug from the PG/ethanol mixture [18]. Open application of 40% EME-containing drug at 43.4 mg/ml produced a flux value of 5.85 μg cm^−2^ h^−1^. This flux is 80% of that recorded after open application of the formulation with the drug being included at saturation (Tables 3).

This finding indicates that supersaturation plays a role in the enhanced transdermal delivery of the progesterone after open application. Comparing this flux recorded after open application of 40% EME containing 2DS with that obtained after open application of EFME, the former was 2.48-fold higher compared to the latter (Table 3). This means that supersaturation is not the only mechanism operating.

Overall, this study suggested a combination of mechanisms. These mechanisms include the penetration-enhancing effect, increased drug partitioning, and possible supersaturation arising from evaporation of volatile components of ME, or phase change due to dilution [2, 6, 9–11]. In addition, the nano-structure and low surface tension can provide a large surface area, and provide excellent contact with the skin surface and its microscopic gaps. This should enhance the vehicle skin-drug transfer [6]. It is clear that these mechanisms can maximally operate in the fluid state of microemulsion. The current study provided an additional finding, highlighting the feasibility of open application. This opens the gate for the introduction of topical sprays in microemulsion transdermal delivery. This mode of application will provide a simpler formulation protocol compared to other strategies, which include the addition of a thickening agent which may alter the efficacy of ME. Carbopol 940 was used to increase the viscosity of ME, but the recorded estradiol transdermal flux was higher in the case of fluid microemulsion compared with the corresponding gelled system [8]. Carrageenan was used to increase the viscosity of microemulsion [20]. Ethyl oleate-based ME was shown to increase the transdermal delivery of ibuprofen. The authors employed xanthan gum to construct the microemulsion-based hydrogel for topical administration. However, the study presented data on the stability of the gel system, but no skin permeation data was presented for the gelled ME [21]. Carbomer-thickened ME enhanced skin permeation, with the enhancement being increased further after incorporation of 1% menthol [22]. Isopropyl myristate-based ME was stabilized with gelatine, which improved skin permeation of cyclosporine [23]. These gel-based systems utilized mainly non-volatile components. This line of research requires further investigation before drawing a final conclusion, as the open application of fluid ME may provide an additional advantage.

In conclusion, the study introduced microemulsion as a potential transdermal delivery system for progesterone. Incorporation of ethanol in the microemulsion system is beneficial. Open application of the microemulsion formulation is possible with the ethanol-containing system, because it retains its advantage even after ethanol evaporation. The enhanced transdermal delivery can be achieved by a combination of mechanisms which include penetration-enhancement, supersaturation, better skin contact, high surface area for drug transfer, and high loading.

## Experimental

### Materials

Progesterone was obtained from E. Merck, Darmstadt. Prednisolone acetate was purchased from Sigma Chemical Co. (St. Louis, MO, USA). Oleic acid was obtained from LOBA Chemie, PVT. LTD, Mumbai, India. Tween 80, acetonitrile (HPLCgrade), ethanol (96%), and propylene glycol were purchased from BDH, England.

### Construction of pseudo-ternary phase diagrams

Oleic acid, a known skin penetration enhancer, was selected as the oily phase. Tween 80 and propylene glycol (4:1) were included as the surfactant and cosurfactant, respectively. These components were used to construct the phase diagram for the basic microemulsion in the absence of ethanol. Propylene glycol (PG) was included to provide an antinucleant effect for the drug after evaporation of ethanol from the ethanol-containing microemulsion. In addition, PG is known to have a synergistic penetration-enhancing effect with oleic acid [19]. To investigate the effect of ethanol on the phase behaviour of the system, pseudo-ternary phase diagrams were also constructed in the presence of different concentrations of ethanol, with ethanol being included as a part of the surfactant-cosurfactant mixture.

The pseudo-ternary phase diagrams were constructed using the water titration method at ambient temperature [6, 24]. “Oil and surfactant/cosurfactant mixtures were prepared at weight ratios of 0.5:9.5, 1:9, 1.5:8.5, 2:8, 2.5:7.5, 3:7, 3.5:6.5, 4:6, 5:5, 6:4, 7:3, 8:2, and 9:1. These were titrated with water under gentile magnetic stirring. The systems were visually characterized after equilibration.” Transparent fluid systems were characterized as microemulsions, but viscous systems that did not show a change in the meniscus after being tilted to an angle of 90° were considered a gel.

### Preparation of tested formulations

The composition of the tested formulations is presented in Table 1. The basic ethanol-free microemulsion formulation (EFME) was comprised of Tween 80, PG, oleic acid, and water (56: 14: 15: 15). This was mixed with ethanol to produce microemulsions containing 20% w/w ethanol (20% EME) or 40% w/w ethanol (40% EME). The microemulsion was prepared by mixing the oil with the surfactant/cosurfactant mixture, before adding the required amount of water under magnetic stirring. Excess progesterone drug was added and equilibrated by continuous mixing in a water bath maintained at 32 °C for 72 hours. This produced saturated drug solutions with excess crystals to maintain saturation. These formulations were used to investigate the effect of the method of application on trans-dermal drug delivery. To investigate the effect of supersaturation, 40% EME was also prepared with 50 mg/ml of the drug. In addition, saturated solutions of the drug were similarly prepared in 40% ethanol/water (ETW) and in 14% PG/water (PGW). The latter was used as the control. No phase change was noted after addition of the drug or after equilibration in the water bath.

### Characterization of the tested formulations

The saturation solubility of the drug in different formulations was determined after being equilibrated at 32 °C for 72 hours. The excess drug was removed by centrifugation and the supernatant was suitably diluted with ethanol before HPLC analysis.

### Ethanol evaporation rate

This test was conducted to approximately predict the rate of ethanol evaporation after open application of the ethanol-containing formulation. The procedures reported by Moser and coworkers were adopted [18]. The microemulsion formulation (40% ETME) was loaded in a glass Petri dish. The amount of formulation was equivalent to that loaded in the donor compartment (volume/area). The weight of the formulation was determined before incubation in an oven maintained at 32 °C. The weight loss was determined periodically. The evaporation profile of the ethanol-free formulation (EFME) was similarly determined and taken as the control.

### Preparation of skin samples

Animal treatment followed the principles in the Declaration of Helsinki. Full-thickness skin obtained from the inner side of freshly excised rabbit ears was used in this study. This model was employed due to the difficulty of obtaining human skin samples, and was found to be successful in monitoring the transdermal delivery of a steroidal drug from ME [6]. The study employed 15 male rabbits, weighing 3.1 ± 0.4 Kg. The skin was peeled from the underlying cartilage after cutting along the tips of the ears. The skin samples were mounted immediately on the diffusion cells.

### Skin permeation studies

The FDC-6 Transdermal Diffusion Cell Drive Console (Logan Instrument Corp., NJ, USA), which was equipped with vertical glass diffusion cells having a diffusional area of 1.7 cm^2^, was used. The skin was mounted with the uppermost side, the stratum corneal [6, 11]. The receptor compartments (12 ml) were filled with 40% v/v propylene glycol in water, which was used as the receptor fluid to ensure sink condition. The same receptor was used to monitor skin delivery of progesterone from microemulsion [25]. The mounted skin was equilibrated overnight with the skin surface being maintained at 32 ± 1 °C. The tested formulations (400 μl) were applied to the surface of the skin, which was left open to the atmosphere (non-occlusive application) or occluded with a parafilm plug lined with aluminum foil (occlusive application). Receptor samples were taken periodically and replaced with fresh receptor fluid. These samples were analyzed for the drug content by HPLC.

To investigate the effect of skin pre-treatment with 40% ethanol in water on transdermal delivery of progesterone from EFME, the equilibrated skin was treated with non-medicated 40% ethanol for 3 hours, at the end of which the donor compartments were dried with a tissue paper. The medicated EFME was loaded into the donor, and the experiment was completed as before.

### Chromatography

The drug content of each sample was determined using a high-pressure liquid chromatograph (Waters^TM^ 600 controller, USA) equipped with an automatic sampling system (Waters^TM^ 717 Plus Autosampler, USA). Detection was performed at 240 nm, using a variable wavelength UV-Vis detector (SPD-10 AV, Shimadzu, Kyoto, Japan). The whole system was controlled by acomputer. Separation employed a reversed-phase column 15 cm × 3.9 mm (i.d.) C18, μ BondapakTM, Waters, with an average particle size of 10 μm. The mobile phase was comprised of acetonitril and water (60:40) flowing at 1 ml/min. Prednisolone acetate was used as the internal standard. The chromatographic data analysis was performed with the Millinium^TM^ Program (Waters, USA).

The receptor samples were transferred to test tubes spiked with the internal standard in an amount sufficient to produce a concentration of 2 μg/ml. These were vortex-mixed for 2 minutes before being loading into HPLC vials and injecting 30 μl into the HPLC.

### Data analysis

The cumulative amounts of the drug permeated with time produced the permeation profiles. These profiles were used to calculate the transdermal drug flux from the slope of the regression line fitted to the linear portion of the profile. Extrapolation of this line will intercept with the x axis at a time equal to the lag time [6, 11].

Student’s *t*-test was used for statistical analysis.

## Figures and Tables

**Tab. 1:** The composition of the tested formulations and drug solubility in each formulation.

Formulation code	Composition	Solubility (mg/ml)
EFME	Tween 80: PG: oleic acid: water (56: 14: 15: 15)	21.7 (0.8)
20% EME	20% ethanol in EFME	48.7 (1.3)
40% EME	40% ethanol in EFME	72.2 (2.6)
ETW	40% ethanol in water	1.43 (0.08)
ETPGW	Ethanol: PG: water (40: 14: 46)	4.33 (0.23)
PGW	14% PG in water	0.176 (0.006)

Values between brackets are SD, n = 3. PG is propylene glycol and ND means not determined.

**Tab. 2:** The transdermal permeation parameters of progesterone obtained after occlusive and non-occlusive application of various formulations.

Formulation	Occlusive application		Open application	
	
	Flux (μg cm^−2^ h^−1^)	Lag time (h)	Flux (μg cm^−2^ h^−1^)	Lag time (h)
EFME	2.69 (0.24)	3.22 (0.34)	2.36 (0.08)	3.41 (0.64)
20% EME	6.20 (0.68)	1.47 (0.64)	4.48 (1.44)	1.50 (0.14)
40% EME	9.83 (2.77)	0.91 (0.41)	7.25 (2.01)	0.28 (0.22)
ETW	2.22 (0.28)	1.88 (0.31)	1.75 (0.25)	0.29 (0.09)
PGW	1.09 (0.18)	2.45 (0.07)	1.34 (0.33)	3.62 (0.75)

Values between brackets are SEM, n = 3.

**Tab. 3:** The transdermal permeation parameters of progesterone obtained after non-occlusive (open) application of various formulations.

Formulation	Open application without skin pretreatment		Open application after skin pretreatment with 40% ethanol	
	**Flux (μg cm**^−^**^2^** **h**^−^**^1^)**	**Lag time (h)**	**Flux (μg cm**^−^**^2^** **h**^−^**^1^)**	**Lag time (h)**
EFME	2.36 (0.08)	3.41 (0.64)	2.85 (0.20)	1.79 (0.12)

	**Open application of fully saturated formulation**		Open application of formulation containing drug at 2 degrees of saturation (DS)*	
	
	**Flux (μg cm**^−^**^2^** **h**^−^**^1^)**	**Lag time (h)**	**Flux (μg cm**^−^**^2^** **h**^−^**^1^)**	**Lag time (h)**
40% EME	7.25 (2.01)	0.28 (0.22)	5.85 (0.12)	0.81 (0.19)

Values between brackets are SEM, n = 3.

* DS of 2 was prepared by dissolving 43.4 mg/ml of drug in 40% EME. Open application of this formulation will create ME with 2 DS after evaporation of ethanol.
